# Quantitative Evaluation of E1 Endoglucanase Recovery from Tobacco Leaves Using the Vacuum Infiltration-Centrifugation Method

**DOI:** 10.1155/2014/483596

**Published:** 2014-05-26

**Authors:** Nathaniel J. Kingsbury, Karen A. McDonald

**Affiliations:** Department of Chemical Engineering and Materials Science, University of California at Davis, 1 Shields Avenue, Davis, CA 95616, USA

## Abstract

As a production platform for recombinant proteins, plant leaf tissue has many advantages, but commercialization of this technology has been hindered by high recovery and purification costs. Vacuum infiltration-centrifugation (VI-C) is a technique to obtain extracellularly-targeted products from the apoplast wash fluid (AWF). Because of its selective recovery of secreted proteins without homogenizing the whole tissue, VI-C can potentially reduce downstream production costs. Lab scale experiments were conducted to quantitatively evaluate the VI-C method and compared to homogenization techniques in terms of product purity, concentration, and other desirable characteristics. From agroinfiltrated *Nicotiana benthamiana* leaves, up to 81% of a truncated version of E1 endoglucanase from *Acidothermus cellulolyticus* was recovered with VI-C versus homogenate extraction, and average purity and concentration increases of 4.2-fold and 3.1-fold, respectively, were observed. Formulas were developed to predict recovery yields of secreted protein obtained by performing multiple rounds of VI-C on the same leaf tissue. From this, it was determined that three rounds of VI-C recovered 97% of the total active recombinant protein accessible to the VI-C procedure. The results suggest that AWF recovery is an efficient process that could reduce downstream processing steps and costs for plant-made recombinant proteins.

## 1. Introduction

Plant-based production platforms can produce a wide variety of functional proteins including cell wall degrading enzymes [1], biopharmaceuticals [2, 3], and vaccines [4]. Advances in* Agrobacterium*-mediated gene transfer [5] have offered a popular and facile way to express these proteins of interest (POI) in a variety of species of plants by both stable transformation methods and through transient expression in wild-type plants in contained facilities [6]. Protein manufacturing in plants has advantages such as low cost of production, scalability, and good mimicry of native human glycosylation [7–9]. However, the cost of recovering and purifying POI from the plant tissue can account for up to 90% of the total cost since alkaloids, phenolics, fiber, proteases, and contaminating proteins all need to be removed for most applications [10, 11].

**Figure 1:**
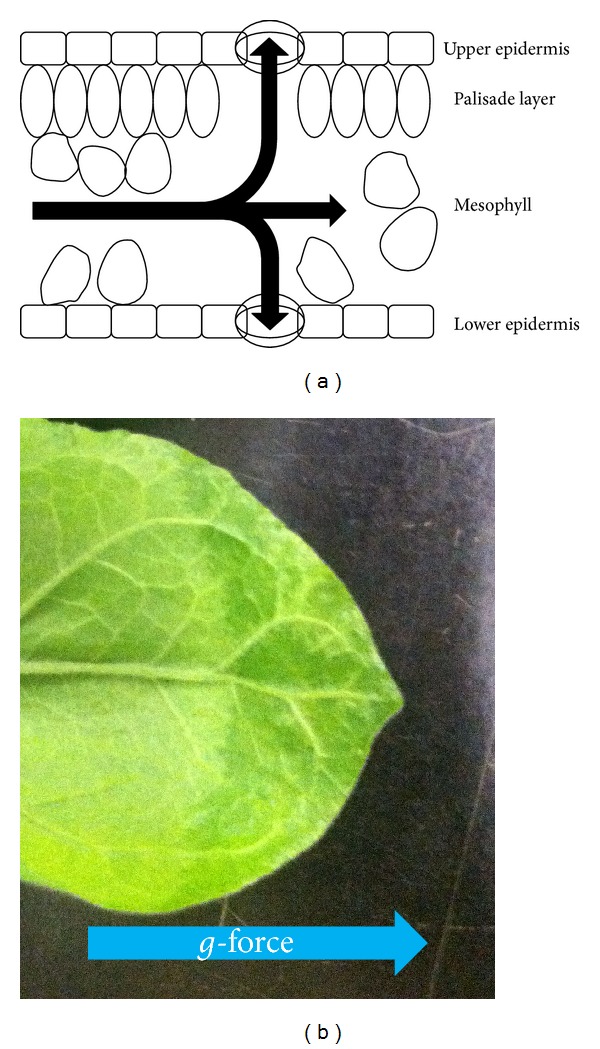
(a) A schematic of the cross-section of a tobacco leaf. Arrows represent the bulk flow of the apoplast wash fluid through the leaf. The flow is in the direction of the centrifugal force as fluid is forced out through the stoma. (b) The centrifugation step to recover AWF from this* Nicotiana benthamiana *leaf was interrupted. The bulk flow of the infiltrated fluid moves evenly through the leaf over time in the direction of the centrifugal force (arrow).

**Figure 2:**
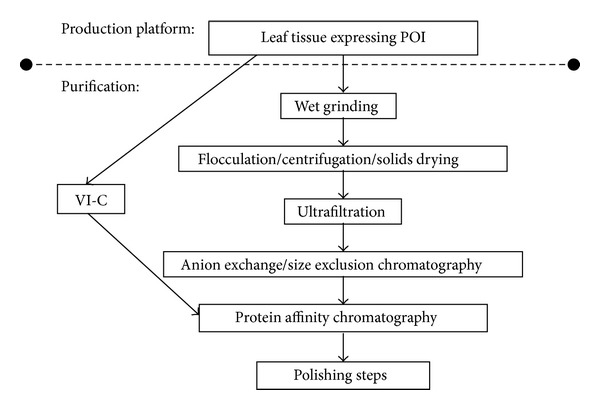
A typical protocol for recovering and purifying recombinant proteins of interest (POI) from leaf tissue, also depicting the steps that could be circumvented by employing the vacuum infiltration-centrifugation (VI-C) method.

These components are present in leaf extracts because they are typically prepared by homogenization, an extraction method performed with buffer that destructively grinds the whole leaf. This process liberates most of the soluble protein in the leaf, but the extract includes not just the POI but also contaminating proteins from every subcompartment of the cell. Fortunately though, understanding of the biological mechanisms of the plant secretion pathway [12] has enabled the engineering of gene constructs which target secretion of POI outside the cells. The dissolved proteins secreted to the aqueous phase of the extracellular space, called the apoplast, allow for techniques that recover POI without breaking open cells. Recovering the apoplast has been used for collecting secreted recombinant proteins for two decades [13], and it continues to be a desirable goal [14, 15]. The vacuum infiltration-centrifugation method (VI-C) is the most common method of recovering the apoplast, and it is potentially feasible at large scales [16, 17]. During the vacuum infiltration step, the leaf is submerged in buffer. Air is pulled through the leaf stoma during vacuum application and the buffer floods the extracellular space after vacuum release, dissolving the apoplast and increasing the overall weight of the leaf tissue [18]. The centrifugation is employed to force this fluid out of the leaf through the stoma, as seen in Figure 1. This restores the leaf to near its initial or fresh weight and yields an extract called the apoplast wash fluid (AWF). The AWF only draws from the extracellular space, so it is already clarified from the vast majority of the contaminants that makes purification from homogenate extracts (HE) so prohibitive. The large number of downstream processing steps required for most industrial applications [6, 10, 15] may be circumvented by switching the recovery method from homogenate extraction to VI-C, as seen in Figure 2.

In this study various efforts to maximize the yields of POI in the AWF by improving the VI-C method was achieved at bench scale. In its origins, VI-C had been used by plant pathologists to study the leaf apoplast as a substrate for the growth of infectious bacteria [19]. It has continued to be used by plant physiologists studying the native composition of the apoplast [20–25]. In such efforts, obtaining concentrated fluid as free as possible from intracellular contaminants is the major goal. However, the technique has only been used to a limited extent as a means for larger scale recovery of recombinant proteins. To learn how VI-C could be applied for maximal recovery of apoplast components, we utilized the colorimetric marker indigo carmine to enable clear visualization of the blue dye before and after AWF recovery. To totally clear any visible indigo carmine from the leaf required applying two additional rounds of VI-C, the increasing centrifugal force to ensure recovery of the greatest volume of AWF, and using three applications of vacuum pressure per round of vacuum infiltration, which maximized buffer uptake by the leaf tissue. For further studies, the selection of stable and nonbinding POI was desired for examining whether expressed secreted proteins in AWF could be recovered as effectively and if not what fraction of the protein was inaccessible to the VI-C technique. For this, a native protein, peroxidase, as well as a thermophilic heterologous protein, E1 endoglucanase from* A. cellulolyticus,* were selected. In the E1 study, both the catalytic domain (E1cd) and the full-length holoenzyme (E1holo, possessing both the cellulose binding domain and the catalytic domain) were transiently expressed in* N. benthamiana* to demonstrate the effect of the additional domain on endoglucanase yield by AWF recovery.

## 2. Materials and Methods

### 2.1. Plant Material

*Nicotiana benthamiana* var. TW16 (National Germplasm Resources Laboratory, Accession #: PI 555478, Beltsville, MD) and* Nicotiana tabacum* var. Xanthii (B. Falk Lab, UC Davis Plant Pathology, Davis, CA) were grown from seed in a greenhouse with a temperature range of 21°C–34°C, on average 28°C ± 3°C, and an observed average absolute humidity of 12 ± 2 g/m^3^. Two weeks after seeding, seedlings were transplanted three in a 6^″^ pot with Sunshine Mix #1 soil (Sun Gro Horticulture, Vancouver, BC). A 6^″^ pot with three five-week-old* N. tabacum *plants were transplanted together a second time into 12^″^ pots. Pots were watered twice a day by an automated irrigation system and a custom fertilizer injection system comprising twelve essential plant nutrients.

Twelve- or thirteen-week-old* N. tabacum* leaves were excised by cutting the petiole from the stem about 30 cm above the soil level and were brought from the greenhouse to the lab in sealed plastic containers kept humid with moist paper towels. Five-week-old* N. benthamiana* plants were brought to the lab whole, and prior to the incubation or agroinfiltration the most recent mature leaves (between the third to the fifth leaf from the meristem) were excised.

Prior to apoplast wash fluid recovery the midrib and edges of each leaf were removed and strips of lamina tissue were excised from the intercostal regions between the veins, strip width not exceeding 1 cm, similar to Rathmell's technique [25]. In some experiments, each leaf was then partitioned into two identical strip sets, in which case one set was selected for apoplast wash fluid recovery (the “washed” set) while the other identical set went unprocessed (the “unwashed” set), incubating in a humidity chamber throughout the experiment until homogenization. Each strip set was cut to weigh 700–800 mg.

### 2.2. E1 Constructs

**Figure 3:**
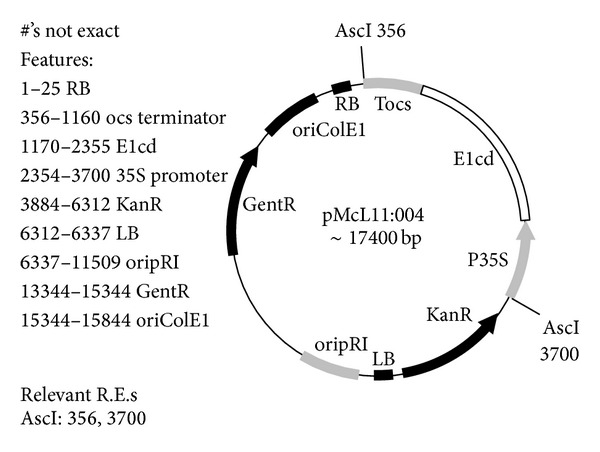
Plasmid pMcL11:004, the binary plasmid for transformation into* A. tumefaciens*, created by cutting the backbone plasmid pDU97:1005 with AscI and insertion of the 35S-E1cd-Tocs gene.

The gene for full-length E1 endoglucanase (E1holo) from* Acidothermus cellulolyticus* (NCBI Accession #: P54583) was codon-optimized using GeneDesigner software (version 1.1.4.1, DNA 2.0, Burlingame, CA) and the codon usage table for* N. benthamiana *[26]. The 41 amino acid native signal peptide was removed from the N-terminus and replaced with the RAmy3D signal peptide from the *α*-amylase gene from* Oryza sativa* (NCBI Accession #: M59351.1). To the C-terminus, a 6-His tag was added. The construct was placed under the control of the CaMV 35S promoter and octopine synthase terminator (Tocs). The sequence and the gene in entirety were submitted to GenBank (Accession #: HQ541433). The method for construction for the truncated E1 (E1cd) was the same as for the full-length protein except with the cellulose binding domain and linker region between domains removed. DNA 2.0 (Menlo Park, CA) synthesized, verified, and inserted the optimized genes for both E1cd and E1holo into pJ201, one of its standard cloning vectors, prior to propagation in* E. coli.* The genes were then ligated into a binary vector, pDU97.1005 (modified by SL Uratsu and AM Dandekar, unpublished, from pCGN1547 [27]) for transformation into* Agrobacterium tumefaciens* EHA105 pCH32 (Figure 3).

### 2.3. Agroinfiltration

Transformed* A. tumefaciens *was thawed from glycerol stocks and grown in small volumes of Luria-Bertani (LB) medium in round-bottom 10 mL tubes at 28°C in an incubator shaking at 250 rpm. Cultures were then inoculated 1% v/v into 200 mL LB medium and incubated again for 18–22 h at 28°C and 250 rpm. After growth, the bacteria were centrifuged for 20 min at 3,200 g. The pellet was resuspended in activation solution consisting of 10 mM 2-(*N*-morpholino) ethanesulfonic acid (pH = 5.6), 10 mM MgCl_2_, and 150 *μ*M acetosyringone (3′,5′-Dimethoxy-4′-hydroxyacetophenone; Aldrich Chemicals, Milwaukee, WI) to an optical density at 600 nm of 0.5, as measured by a SpectraMax M2 spectrophotometer (Molecular Devices, Sunnyvale, CA). In the activation solution, the bacteria cultures were incubated in the dark for two to five hours and just prior to agroinfiltration 0.02% v/v Silwet L-77 (Lehle Seeds, Round Rock, TX) was added.

Detached plant leaves were held submerged into activated* Agrobacterium* solution in a plastic container by a plastic lined wire mesh. Three rounds of vacuum infiltration were applied in a 5-gal Nalgene vacuum chamber, where for each round the pressure was allowed to reach an absolute pressure 30 kPa held at that pressure for at least 30 s. Leaves were then patted dry with paper towels and allowed to dry on a rack for an hour prior to incubation.

### 2.4. Incubation

Incubation was performed in a 19^″^ × 14^″^ × 7^″^ air-tight plastic storage box. Perlite soil additive (E.B. Stone Organics, Suisun City, CA) was submerged in DI water for at least three hours and poured into the box to create a layer about 4 cm thick to maintain humidity throughout the incubation. Plastic lined steel mesh was fit into the box to suspend the leaves about 4 cm above the Perlite layer. The box with detached leaves was incubated in the dark at 20°C.

### 2.5. Apoplast Wash Fluid Recovery

Plant strips were rinsed to remove any debris from their surfaces, blotted dry with paper towels, and weighed to obtain their fresh weight. Strips were then placed in 50 mL Falcon tubes and submerged in 20°C–25°C harvest buffer at a buffer to biomass ratio ranging from 10 to 25 mL/g FW. The harvest buffer consisted of 50 mM sodium acetate (pH = 5.5), 100 mM NaCl, and 0.02% Silwet L-77. In the indigo carmine experiments, leaf strips were vacuum infiltrated with 1900 *μ*g/mL indigo carmine (Sigma, St. Louis, MO), dissolved in 50 mM sodium acetate (pH = 6.2), 100 mM NaCl, and 0.02% Silwet L-77. Three rounds of vacuum application were performed in a Nalgene container and brought to an absolute pressure of 30 kPa and held for 30 s prior to release for at least 30 s between rounds. Strips were removed from the buffer, and the buffer in the tubes was capped, refrigerated, and kept as “Rinse Fluid” (RF) samples. Strips were blotted dry with paper towels and weighed again to monitor the volume of buffer infiltrated into the strips.

Vacuum infiltrated leaf strips were loaded into perforated 50 mL Falcon tubes with no particular orientation and centrifuged to obtain the AWF. Each Falcon tube possesses 8–15 circular perforations about 3 mm in diameter each. The perforated tubes with the leaf strips were transported to the centrifuge in a humid box. It was found that fixed angle centrifuges damaged the leaf strips unacceptably, observed as green coloration in the AWF, so a Beckman GS-6KR (Beckman Coulter, Inc., Brea, CA) centrifuge with swing-out wells was used. Collection caps were fashioned from the bottom halves of 50 mL Falcon tubes and they were positioned in the centrifuge under the perforated tubes to catch the recovered AWF. The centrifuge was run at 25°C for 10 minutes at 3,200 g, and the AWF was recovered from the collection cap, the volume was measured and recorded, and the AWF sample was refrigerated prior to assaying performed the same day. The perforated tubes with the centrifuged leaf strips inside were brought back to the lab in a humid box and the new tissue weight was measured prior to the next round of vacuum infiltration. Vacuum infiltration and centrifugation steps of subsequent rounds were performed the same as the first, and changes in the weight of the strips were monitored throughout the experiment (Supplemental Figure 1 in Supplementary Material available online at http://dx.doi.org/10.1155/2014/483596).

### 2.6. Homogenate Extraction

Leaf strip sets were homogenized with liquid nitrogen or wet grinding after each experiment. Powder from liquid nitrogen extraction was resuspended in 10 mL/g FW ice cold harvest buffer in a 15 mL Falcon tube. The powder was allowed to incubate in the tube in an ice bath for ten minutes prior to centrifugation for 10 min at 4°C and 6,000 g. Exactly 1 mL of the supernatant was decanted into 1.5 mL Eppendorf tubes and centrifuged again for 20 min at 4°C and 20,000 g (Eppendorf Centrifuge 5403, Hauppauge, NY) prior to assaying. For the results in the E1cd and E1 holoenzyme studies, a GrindoMix GM 200 (Retsch Technology, Haan, Germany) was used to homogenize leaf strip sets. Strips were homogenized with 50 mL of ice cold harvest buffer at 8,500 rpm for two rounds of 15 s each.

### 2.7. Quantitative Analysis

Indigo carmine was dissolved in 50 mM sodium acetate 100 mM NaCl and 0.02% Silwet L-77. A linear standard curve was developed from 0–333 *μ*g/mL indigo carmine by direct measurement of 50 *μ*L per well at an absorbance of 608 nm by a SpectraMax 340pc (Molecular Devices, Sunnyvale, CA).

The total soluble protein assay was performed by the method of Bradford [28] using Coomassie Brilliant Blue G-250 dye (Bio-Rad, Hercules, CA). A standard curve was produced from bovine serum albumin (BSA) (Fisher, Pittsburgh, PA) diluted in harvest buffer. Sample, diluted sample, or standard measuring 10 *μ*L was added to 90 *μ*L harvest buffer in a 96-well plate. Bradford dye measuring 200 *μ*L was added to each well and color was developed for five minutes prior to the measurement of absorbance at 590 nm by a SpectraMax 340pc.

Malate dehydrogenase (MDH) activity assay for measurement of intracellular contamination in apoplast wash fluid or rinse fluid was performed as described [24, 29]. A standard curve was produced from 0.75 mM *β*-nicotinamide adenine dinucleotide, reduced dipotassium salt (NADH) (Sigma-Aldrich, St. Louis, MO) diluted in 50 mM phosphate buffer (pH = 7.5), 200 *μ*L per well. Then, 10 *μ*L sample or diluted sample was added to 90 *μ*L phosphate buffer in a 96-well plate at room temperature. The reaction was started when 50 *μ*L 1.5 mM and 50 *μ*L of 2 mM oxaloacetic acid (OAA) (Sigma-Aldrich, St. Louis, MO) were added to each sample or diluted sample well. The decrease in absorbance at 340 nm in the sample wells, corresponding to the conversion of NADH to NAD^+^ by MDH in a reversible redox reaction that also converts OAA to malate, was monitored for three minutes and compared to the NADH standard curve by a SpectraMax 340pc.

Peroxidase activity was measured by monitoring the conversion of pyrogallol to purpurogallin in the presence of hydrogen peroxide [30]. Horseradish peroxidase powder (Sigma, St. Louis, MO) dissolved to 1.5 Units per mL was used to generate a standard curve. To each well of a 96-well plate, 10 *μ*L of sample, diluted sample, or horseradish peroxidase standard was added to 190 *μ*L of 200 mM KH_2_PO_4_ (pH = 6.0) buffer. A 1 : 1 mixture of 0.3% hydrogen peroxidase and 50 mg/mL pyrogallol were prepared just before assaying and 100 *μ*L was added to each well. Change of absorbance at 420 nm corresponding to the production of purpurogallin was measured over a 40 s duration by a SpectraMax 340pc.

The activity of E1 endoglucanase was measured fluorometrically using methylumbelliferyl-*β*-D-cellobioside (MUC) as a substrate as described previously [31, 32]. E1 converts the MUC substrate, which is not fluorescent, to 4-methylumbelliferone (MU), and 3 *μ*M MU diluted in acetate buffer (50 mM acetate, 100 mM NaCl, pH = 5.5) was used to generate a standard curve. Samples were diluted to 600 *μ*L in acetate buffer and incubated with 200 *μ*L of 500 *μ*M MUC or 125 *μ*M MUC in 1.5 mL Eppendorf tubes at 65°C for 30 min. Before and after the reaction, 200 *μ*L of the reaction volume was transferred to 800 *μ*L stop buffer (150 mM glycine buffer, pH = 10.0) and the change in fluorescence (*λ*_ex_ 360 nm/*λ*_em_ 460 nm) was measured with a VersaFluor fluorometer (Bio-Rad, Hercules, CA). As an alternative to using the VersaFluor, some samples were read by adding 50 *μ*L of reaction volume to 50 *μ*L stop buffer in triplicate wells of an opaque 96-well plate and read with a SpectraMax M2 (Molecular Devices, Sunnyvale, CA). A unit of activity (U) was defined as the generation of one nanomole of MU per minute [32]. Recombinant E1cd has been previously purified from* Streptomyces lividans* [33] and its specific activity has been previously reported at 40 U per microgram [32]. E1holo is estimated to have a specific activity of 20 U per microgram based on assuming that it has the same molar specific activity as E1cd and accounting for the ratio of their molecular weights.

### 2.8. Calculations

The percent yield of a component may be calculated by the formula

(1) $$\begin{array}{c} \%\;\text{yield}=100\%*\frac{\sum_{i=1}^{n}{\text{AWF}}_{i}+\sum_{i=1}^{n}{\text{RF}}_{i}}{\text{total}}, \end{array}$$

where the “total” is the amount of the component in the leaf prior to recovery, assumed to be the sum of the yields from all the AWF and all the RF from every round of recovery as well as the yield in WHE (the homogenized tissue from which the AWF had been recovered).

The purity fold improvement of a component calculated for a given sample (AWF, RF, or WHE) was calculated as the ratio of its specific yield relative to the specific yield in the total. The concentration fold improvement of a component in a sample was calculated by dividing that component's concentration in the sample by that of the total, standardizing it to a 10 mL/g FW buffer to biomass ratio. The purity and concentration fold improvements in volume *V* of component *k* can be given as follows:

(2) $$\begin{array}{c} \text{Purity}\;\;\text{fold}\;\;\text{improvement}=\frac{{\left[k\right]}_{V}/{\left[\text{TSP}\right]}_{V}}{{\left[k\right]}_{\text{total}}/{\left[\text{TSP}\right]}_{\text{total}}},\\ \text{Concentration}\;\;\text{fold}\;\;\text{improvement}=\frac{{\left[k\right]}_{V}}{{\left[k\right]}_{\text{total}}}. \end{array}$$

In indigo carmine experiments, the dilution factor (*F*_dil_) was calculated to make predictions for how much the product would be diluted in subsequent washes. It describes the ratio between total extracellular space and the volume of the liquid apoplast phase to show theoretically how much an apoplast component would be diluted per round of vacuum infiltration and AWF recovery (3). The volume of the apoplast can be measured by infiltrating a leaf with a marker, *k*, impermeable to cell membranes (such as indigo carmine) and observing the decrease in its concentration from the infiltration solution to the recovered AWF (4) [34]. An *n*-number of rounds of vacuum infiltration-centrifugation washes is theorized here to have an exponential effect on the concentration of the marker in the *n*th recovered yield (5). Equation (5) assumes that there are no changes in the volumes of either the total leaf extracellular volume or its apoplast between rounds of recovery. It assumes complete mixing of the infiltrated buffer with the latent liquid phase prior to centrifugation, and it also assumes the marker or POI is completely soluble and does not interact with the extracellular matrix. Equation (5) does not account for loss of the component into the RF during the vacuum infiltration step, so experimentally observed yields were calculating by adding the content in the AWF with the content in the RF during comparison with model predictions. Equation (6) describes the maximum yield from the summation of theoretically infinite rounds of VI-C:

(3) $$\begin{array}{c} F_{\text{dil}}=\frac{{V_{\text{air}}+V}_{\text{apo}}}{V_{\text{apo}}}, \end{array}$$

(4) $$\begin{array}{c} V_{\text{apo}}=V_{\text{air}}*\frac{{\left[k\right]}_{\text{buffer}}}{{\left[k\right]}_{\text{AWF}}}-V_{\text{air}}, \end{array}$$

(5) $$\begin{array}{c} {\text{Yield}}_{n}=\frac{{\text{Yield}}_{1}}{F_{\text{dil}}^{n-1}}, \end{array}$$

(6) $$\begin{array}{c} \overset{\infty }{\underset{n=1}{\sum }}{\text{Yield}}_{n}={\text{Yield}}_{1}*\frac{F_{\text{dil}}}{F_{\text{dil}}-1}. \end{array}$$

Statistical analysis was performed using Microsoft Excel software. Standard deviations reported throughout this paper were calculated just from sample-to-sample variability as other possible sources of uncertainty such as assaying, volume, or leaf weight measurements were considered negligible compared to this. Whether values were statistically different was determined using two-tailed paired student's *t*-tests using a 95% confidence interval as the threshold for significance.

## 3. Results and Discussion

### 3.1. Recovery of Indigo Carmine

Indigo carmine dye was very thoroughly removed from the* N. tabacum* leaf tissue after three rounds of vacuum infiltration-centrifugation. The first AWF recovered from the tissue infiltrated with indigo carmine was dark blue, visually very similar in shade to the infiltration buffer. Subsequent rounds of AWF recovery on the same tissue produced increasingly dilute extracts. Furthermore, the tissue was losing the blue hue it acquired after its infiltration with the dye, becoming indistinguishable from a control set infiltrated with clear buffer by the third round of VI-C.

**Figure 4:**
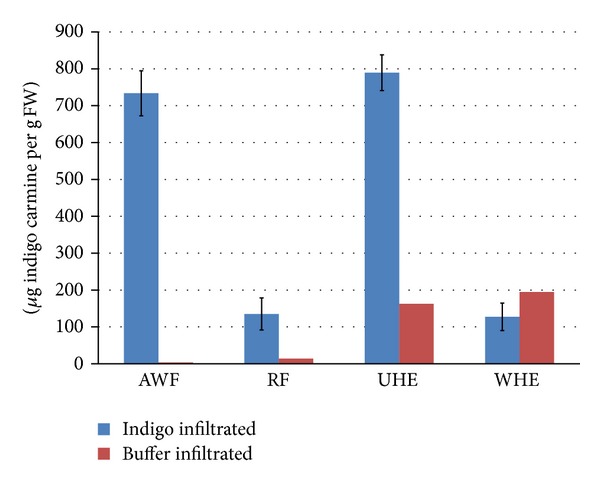
Yields in various extracts from 12-week-old* N. tabacum* leaf strips vacuum infiltrated with 1900 *μ*g/mL indigo carmine (*n* = 3). Chlorophyll produces the background absorbance observed in buffer infiltrated samples (*n* = 1). AWF = apoplast wash fluid; RF = rinse fluid; UHE = unwashed homogenate extract; WHE = washed homogenate extract.

The visual observations were confirmed by analysis of the samples' absorbance at 608 nm and plotted on Figure 4. The absorbance of the indigo carmine in the homogenate from tissue extracted prior to AWF recovery (unwashed homogenate extract; UHE) was much higher than the background from chlorophyll, whose absorbance was observed in the UHE of clear buffer infiltrated tissue. Meanwhile, after three rounds of AWF recovery, the washed homogenate extract (WHE) of the indigo infiltrated leaf tissue had an absorbance that was statistically indistinguishable from the WHE of clear buffer infiltrated leaves (which in turn had a similar absorbance value to the same control's UHE). Meanwhile, the amount of dye recovered in the AWF and the RF was approximately equal to the indigo content in the UHE, determined after adjusting the absorbance reading against background absorbance. These results suggested almost complete recovery of the infiltrated indigo carmine from the tobacco leaf strips in this experiment.

**Figure 5:**
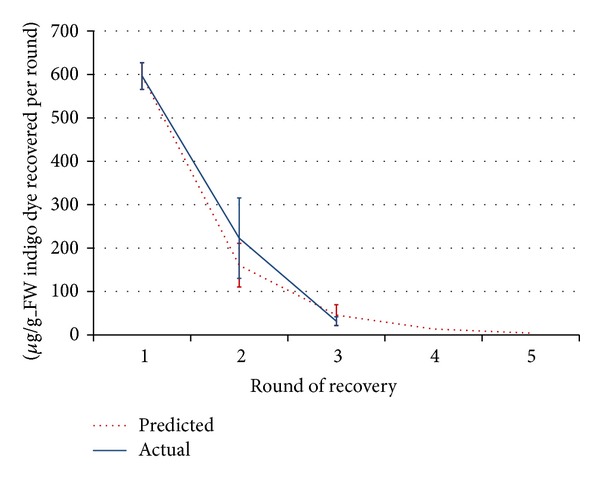
Yields per round of recovery (AWF and RF combined) of infiltrated indigo carmine from 12-week-old* N. tabacum *leaf strips (*n* = 3), showing the experimental results alongside the results predicted from the first round of recovery. Only three rounds of recovery were performed in experiment, while predicted results are shown extrapolated for additional rounds. Error bars in the predicted data reflect variation in* N. tabacum *dilution factors that were observed.

The concentration of the apoplast component recovered during each round of VI-C was demonstrated to be predictable, as seen in Figure 5. By performing one round of AWF recovery, the dilution factor (*F*_dil_) was calculated in this experiment to be 4 ± 1 (*n* = 3), and this value was used to predict the dilution of indigo carmine in AWF recovered in subsequent rounds. The dilution of indigo in these later AWF extracts was more than expected by the model because of the dye leaching from the leaf interstitial space into the acetate buffer during vacuum infiltration. However, by pooling the yield of AWF with the yield of its corresponding RF, the actual observed recovery of indigo showed no statistical difference with predicted values, and so the data was interpreted as in support of the model.

Using the model and (6), it was postulated that for the *F*_dil_ found for this experiment, 97.5% of the total theoretically recoverable indigo carmine was obtainable in three rounds of VI-C. Infinitely more rounds of VI-C would have only improved yields of indigo carmine by an additional 2.5%, so future experiments were limited to three rounds.

### 3.2. Recovery of Peroxidase

**Figure 6:**
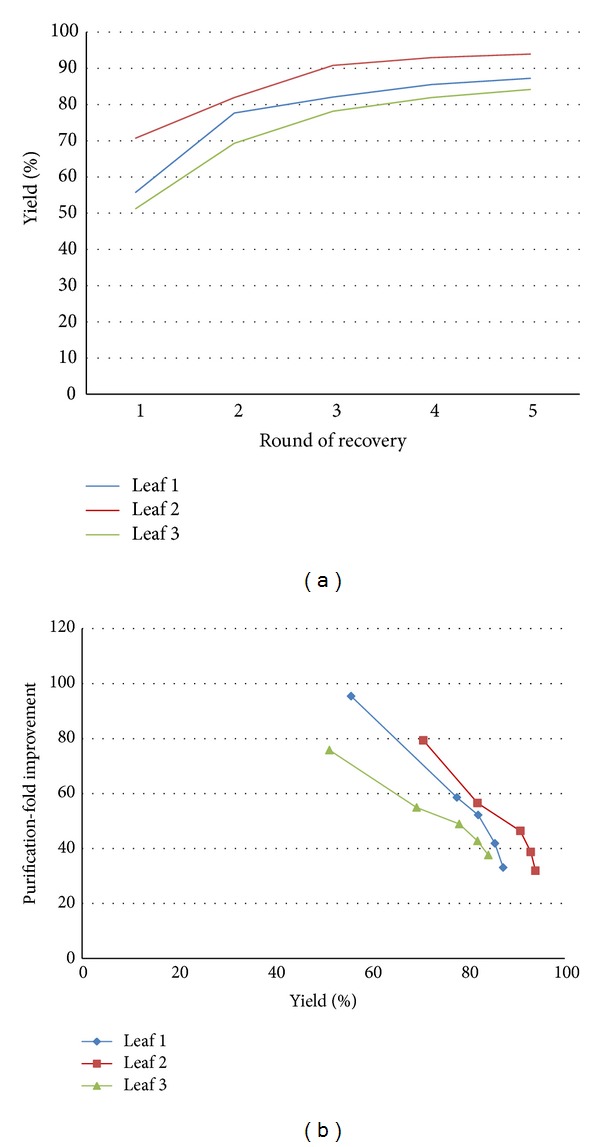
The accumulated percent yield and pooled purification-fold improvement of peroxidase recovered in RF and AWF from tissue excised from triplicate 12-week-old* N. tabacum* leaves over five rounds of vacuum infiltration-centrifugation. (a) Accumulated percent yield after each round of recovery. (b) Purity versus yield chart over five rounds of recovery, depicted for each leaf sample.

Five rounds of VI-C performed on strips of* N. tabacum* leaves in triplicate showed that up to 94% of the peroxidase activity could be recovered in AWF (19, 2.6, and 1.4 U/g FW measured in the accumulated AWF, accumulated RF, and residual homogenate extract resp.) (Figure 6(a)). Up to 71% of the activity was recovered during the first round of recovery and activity was up to 16 times more concentrated than in homogenate extracts (37 versus 2.3 U/mL, normalizing to a buffer to biomass ratio of 10 mL per g FW). Average yields and concentrations during subsequent rounds of average were diluted by a factor of 4, in agreement with the dilution factor of exogenously infiltrated indigo carmine.

Despite the high yields of peroxidase, only a very small percentage of the total soluble protein was recovered, about 2%. While the peroxidase activity was diluted upon each additional round of recovery, TSP levels remained relatively stable. Therefore, while the first recovered AWF and RF had up to 95-fold improvement in purity over homogenate extracts (400 versus 4.2 U/mg TSP), this value decreased as more extracts from the same tissue were collected (Figure 6(b)). The malate dehydrogenase recovered throughout the experiment for each tissue sample was <1%, and since MDH is found typically only in the cytosol or intracellular organelles, this underscores that recovery was highly selective for secreted proteins.

### 3.3. Recovery of Recombinant E1 Catalytic Domain

From* N. benthamiana *tissue agroinfiltrated to express the truncated catalytic domain (E1cd), apoplast wash fluid (AWF) and rinse fluid (RF) samples were collected by three rounds of vacuum infiltration-centrifugation. The total E1cd activity was 2800 ± 1300 units (70 ± 33 *μ*g, based on estimated specific activity) per kg fresh weight of leaf tissue, of which only 23% ±4% remained residually in the WHE (see Supplemental Table 1). In contrast, the WHE retained 86% ±4% of the total soluble protein and 98% ±1% of MDH. As a result of this selective recovery, VI-C produced an extract that is 9 ± 4 times more pure than an extract produced by homogenization.

More than half of the total E1cd activity, 61% ±14%, was recovered during the first round of VI-C, followed by 13% ±4% in the second round and 4% ±1% in the third (See Supplemental Table 2). The observed dilution factor averaged across the three rounds of recovery was therefore 4 ± 1, similar to the result calculated for* N. tabacum *in the indigo carmine and peroxidase studies, and suggested another 4% of the expressed E1cd activity may have been recoverable from additional rounds of recovery. It is therefore predicted of the E1cd expressed, 18% was in regions inaccessible to AWF recovery such as inside the cells or in between tightly packed epidermal cells.

**Figure 7:**
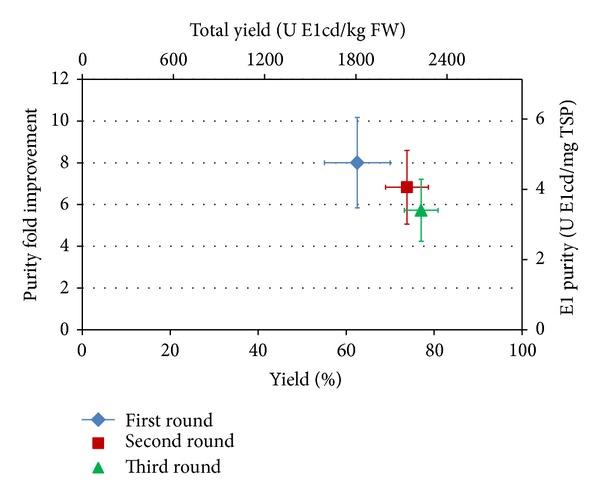
Average accumulated recovery of truncated E1 collected after each of three rounds of infiltration-centrifugation (pooled AWF and RF). Purity is graphed as an improvement over the total expression on one axis and its purity per milligram of total soluble protein on the other axis. Yield is graphed both as percent yield and also as the units of enzyme recovered per kg fresh weight of leaf tissue.

Recovering recombinant protein using consecutive rounds of VI-C may or may not be desirable because a decrease in purity is observed alongside an increase in yield. For each round of recovery the specific activity, or purity, of E1cd went down, dropping from an 8-fold improvement to a 6-fold improvement when the three AWFs and three RFs were combined as seen on Figure 7. When considering just the AWFs, which almost always had the higher specific activity compared to the corresponding RF while also being by far more concentrated, the purity still dropped from a 9- to a 7-fold improvement. The drop in purity between rounds was because E1cd yield was dramatically diluted while the amount of intracellular contamination, as measured by MDH yields, was stable between rounds of recovery. The trend for TSP recovery was in between these two extremes: while the first round yielded significantly more TSP than any subsequent round, after that there were no distinguishable differences (see Supplemental Tables 1 and 2). This underscored the diversity of the leaf's native apoplast proteins, which would have a range of sizes, solubility, and affinity to the cell wall. Since percent yield in the recovered AWF of TSP was always at least three times greater than that for MDH (in the RF, MDH was too diluted to be accurately assayed), it is also concluded that most of the contaminating proteins in these extracts were apoplast localized. In addition to native apoplast proteins, the purity of E1cd may also be negatively affected by the presence of secreted inactive E1cd. Since in these studies E1cd was quantified only by its activity, any inactive E1cd would be included in the overall TSP levels.

### 3.4. Recovery of Recombinant E1 Full-Length Enzyme

**Figure 8:**
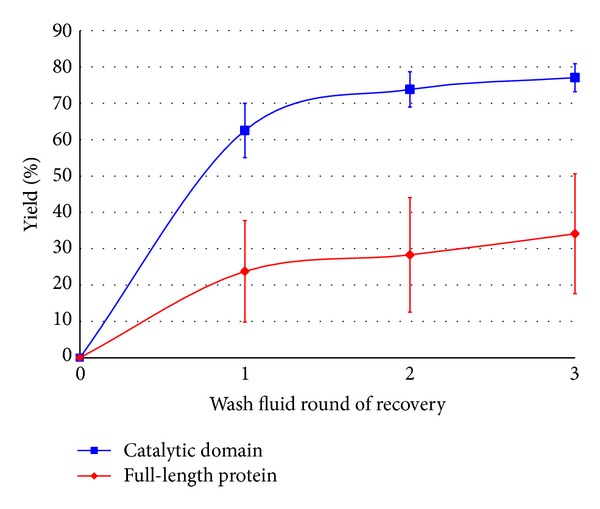
The accumulated recovery (combined AWF and RF) of truncated E1 (catalytic domain) and the full-length E1 endoglucanase after each round of infiltration-centrifugation. Differences in yield between catalytic domain and full-length protein were significant at each round.

The holoenzyme of E1 endoglucanase (E1holo) has a cellulose binding domain, which increases its affinity for the cell wall matrix while also making it substantially bigger than the truncated version (126 kDa versus 51 kDa as visualized on a Western blot, not shown). Because of this, it was expected that the percent yield of the full-length enzyme would be less than that of the truncated isoform. This hypothesis was supported by the experimental results, as seen in Figure 8. The percent yield from three rounds of VI-C was 34% ±17%, substantially less than for the truncated version, which was 77% ±4%. The values for TSP and MDH obtained and their trend over multiple rounds of VI-C however largely matched the results obtained for the sample set expressing E1cd, enabling a fair comparison between sample sets based on the characteristics of the recombinant proteins expressed.

Besides percent yield, there were other differences between the two sample sets. There was an overall lower expression level of E1holo versus E1cd, and it is possible that this would have a negative effect on the percent yield recovered as well. The theory that the cell wall impedes flow of the larger enzyme however is supported by how the dilution factor for this experiment could not even be calculated because in some samples the yields from the third round of VI-C were actually higher than those from the second round. This could be explained by considering the effect of applying centrifugal force on the tissue repeatedly during the course of the experiment, and how this might compromise cell wall integrity and increase its effective porosity. Also, recovery of E1holo, as seen from its relatively larger error bars in Figure 8, was more prone to sample variability, perhaps suggesting that free movement of large recombinant proteins through the apoplast is more sensitive to subtle differences in cell wall morphology. Therefore, it is theorized that the differences observed comparing the recovery of the E1holo and its truncated version were in contrast because of differences in how they interact with the cell wall matrix as AWF is pulled out of the leaf tissue.

## 4. Conclusions

By implementing measures to maximize the amount of buffer infiltrated into the tissue and performing centrifugation until the tissue is restored to its fresh weight, we show for the first time that three rounds of VI-C are sufficient for recovering almost all of the activity of secreted proteins into AWF extracts characterized by remarkable purity and concentration improvements. Over 90% of native peroxidase was recovered from mature wild-type* N. tabacum *leaf strips. From agroinfiltrated* N. benthamiana *leaves, as much as 81% of the expressed E1cd was recovered. Analysis of the dilution factor suggests that recovery of the residual activity within extracted leaf tissue was impeded either by incomplete secretion or by interaction with the cell wall matrix.

These were the highest yields ever achieved from the apoplast of leaf tissue using a nondestructive extraction technique, demonstrating that, if the experiments were repeated at a higher scale, they may be employed to increase extract purity and concentration without enduring major losses in yield. Preliminary tests could be performed with industrial vacuum chambers and centrifuges to scale up recovery of exogenous infiltrated chemicals or native secreted proteins from kilograms of wild-type plant tissue, and knowledge gained from these efforts would directly apply to recovery of heterologous proteins from agroinfiltrated or transgenic tobacco. To conform to the bench scale experiments, the applied vacuum would need to result in the saturation of the leaf tissue with infiltration buffer and the centrifuge step would need to restore the tissue to its original fresh weight without damaging it. As long as these conditions are met, we hypothesize that the AWF extracted at large scale will be relatively pure and enriched compared to conventional large scale homogenization methods.

Other future work explores the use of cell wall degrading enzymes to treat the leaf prior to AWF recovery to improve the percent yield of larger recombinant proteins. Another technique we are developing employs AWF recovery periodically over the entire transient expression phase. This method has the potential to further improve AWF purity while also increasing overall yields of protein per unit weight of leaf tissue.

## Supplementary Material

Included in the supplementary material is mean data for each extract (the WHE, the UHE, and the AWF and the RF) from the experiments recovering peroxidase (Supplementary Table 1), E1cd (Supplementary Table 3), and E1holo (Supplementary Table 5). Also provided are tables for these respective experiments with the mean values for the POI percent yield, purity, and concentration of each extract.
The supplementary material also includes a figure showing the change in weight of the leaf tissue in the indigo carmine and peroxidase experiments during the course of three rounds of VI-C. The weight of the leaf tissue increases and decreases as fluid is moved into and out of its intercellular space.

## Nomenclature

AWF: apoplast wash fluid
E1cd: the catalytic domain of E1 endoglucanase from* A. cellulolyticus*
E1holo: the full-length holoenzyme of E1
*F*_dil_: dilution factor
HE: homogenate extract
POI: protein of interest
RF: rinse fluid, spent vacuum infiltration buffer
TSP: total soluble protein
UHE: unwashed homogenate extract (no VI-C performed on tissue)
VI-C: vacuum infiltration-centrifugation
WHE: washed homogenate extract (tissue after rounds of VI-C).
