# Supplementary material for: Quantitative Evaluation of E1 Endoglucanase Recovery from Tobacco Leaves Using the Vacuum Infiltration-Centrifugation Method
Source: Biomed Res Int. 2014 May 26;2014:483596. doi: 10.1155/2014/483596 (PMC4058203; doi:10.1155/2014/483596)
Supplement: Supplementary file 1 — Included in the supplementary material is mean data for each extract (the WHE, the UHE, and the AWF and the RF) from the experiments recovering peroxidase (Supplementary Table 1), E1cd (Supplementary Table 3), and E1holo (Supplementary Table 5). Also provided are tables for these respective experiments with the mean values for the POI percent yield, purity, and concentration of each extract. The supplementary material also includes a figure showing the change in weight of the leaf tissue in the indigo carmine and peroxidase experiments during the course of three rounds of VI-C. The weight of the leaf tissue increases and decreases as fluid is moved into and out of its intercellular space. [file 483596.f1.docx]

**SUPPLEMENTAL TABLES and FIGURES**

**Supplemental Table I.** Yields from *N. benthamiana* tissue transiently expressing the E1 catalytic domain from *Acidothermus cellulolyticus*

**Supplemental Table II.** Purity and concentration fold improvements and percent yields from *N. benthamiana* tissue transiently expressing E1 catalytic domain from *Acidothermus cellulolyticus*

**
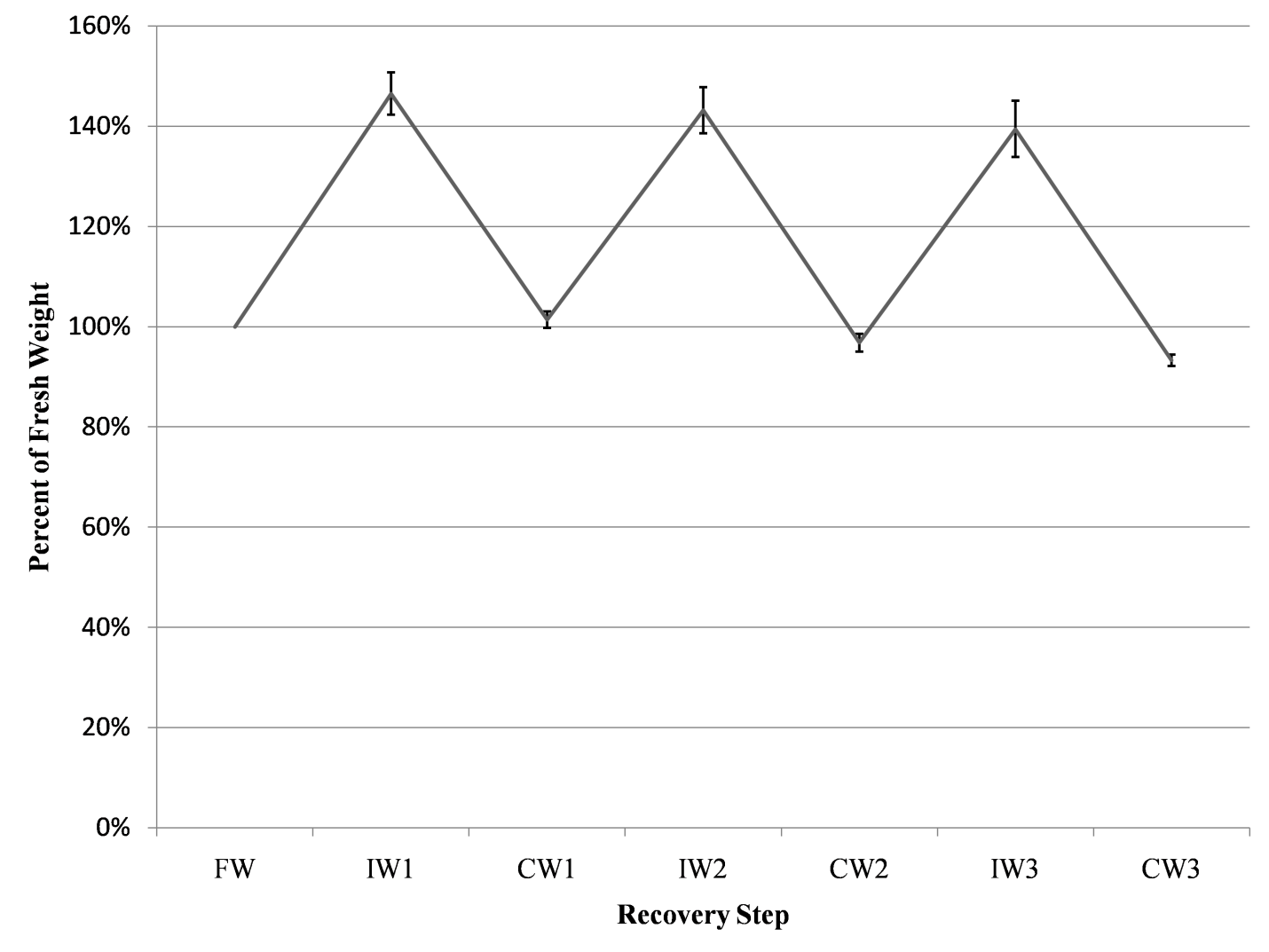
**

**Supplemental Figure 1.** Monitoring weight changes in the *Nicotiana tabacum* leaf strips during the experiments during the indigo carmine experiment. Weight measurements for each strip set was normalized to 100% of its initial fresh weight (FW) and the percentage change from the FW at each step of the three round VI-C recovery process was recorded in both experiments. Results from Sections 3.3 and 3.4 are not reported. IW=Infiltrated weight; CW=centrifuged weight. For section 3.1, n=4 including the control set infiltrated with buffer without indigo carmine. For section 3.2, n=3
